# Supplementary material for: Reward uncertainty asymmetrically affects information transmission within the monkey fronto-parietal network
Source: Commun Biol. 2020 Oct 21;3:594. doi: 10.1038/s42003-020-01320-6 (PMC7578031; doi:10.1038/s42003-020-01320-6)
Supplement: Supplementary file 1 — Supplementary Information [file 42003_2020_1320_MOESM1_ESM.pdf]

Title: *Reward uncertainty asymmetrically affects information transmission within the monkey fronto-parietal network*

Authors: Bahareh Taghizadeh[1,2*], Nicholas C. Foley[3,4*], Saeed Karimimehr[1], Michael Cohanpour[3,4], Mulugeta Semework[3,4], Sameer A. Sheth[5], Reza Lashgari[1] and Jacqueline Gottlieb[3,4,6]

*Equal contribution

Author affiliation: [1]Brain Engineering Research Center, Institute for Research in Fundamental Sciences, Tehran 19395-5746, Iran, [2]School of Cognitive Sciences, Institute for Research in Fundamental Sciences, Tehran 19395-5746, Iran , [3]Department of Neuroscience, Columbia University, [4]Zuckerman Mind Brain Behavior Institute, Columbia University, [5] Department of Neurosurgery, Baylor College of Medicine, [6]The Kavli Institute for Brain Science, Columbia University

Corresponding author:

Jacqueline Gottlieb, PhD

Department of Neuroscience

Columbia University

3227 Broadway,

New York, NY 10025

E-mail: jg2141@ columbia.edu

Fig. S1: Recording sites.

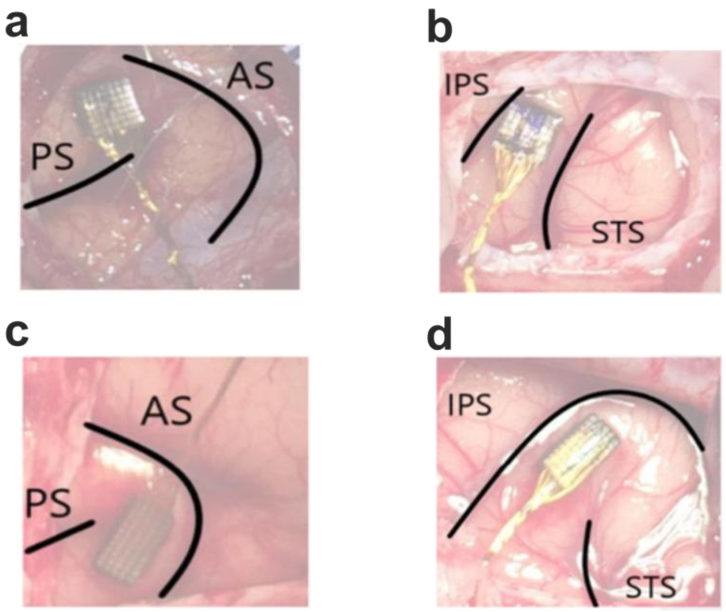

Intraoperative photographs showing array placements. (**a** and **c**) the dlPFC arrays were implanted between the arcuate sulcus (AS) and the principal sulcus (PS), slightly more dorsal in monkey 1 relative to monkey 2 because of vascular anatomy. (**b** and **d**) the 7A arrays were implanted between the intraparietal sulcus (IPS) and superior temporal sulcus (STS), in the posterior portion of this area labeled OPT.

Fig. S2: Variance and EV coding are independent of visuo-spatial selectivity

32

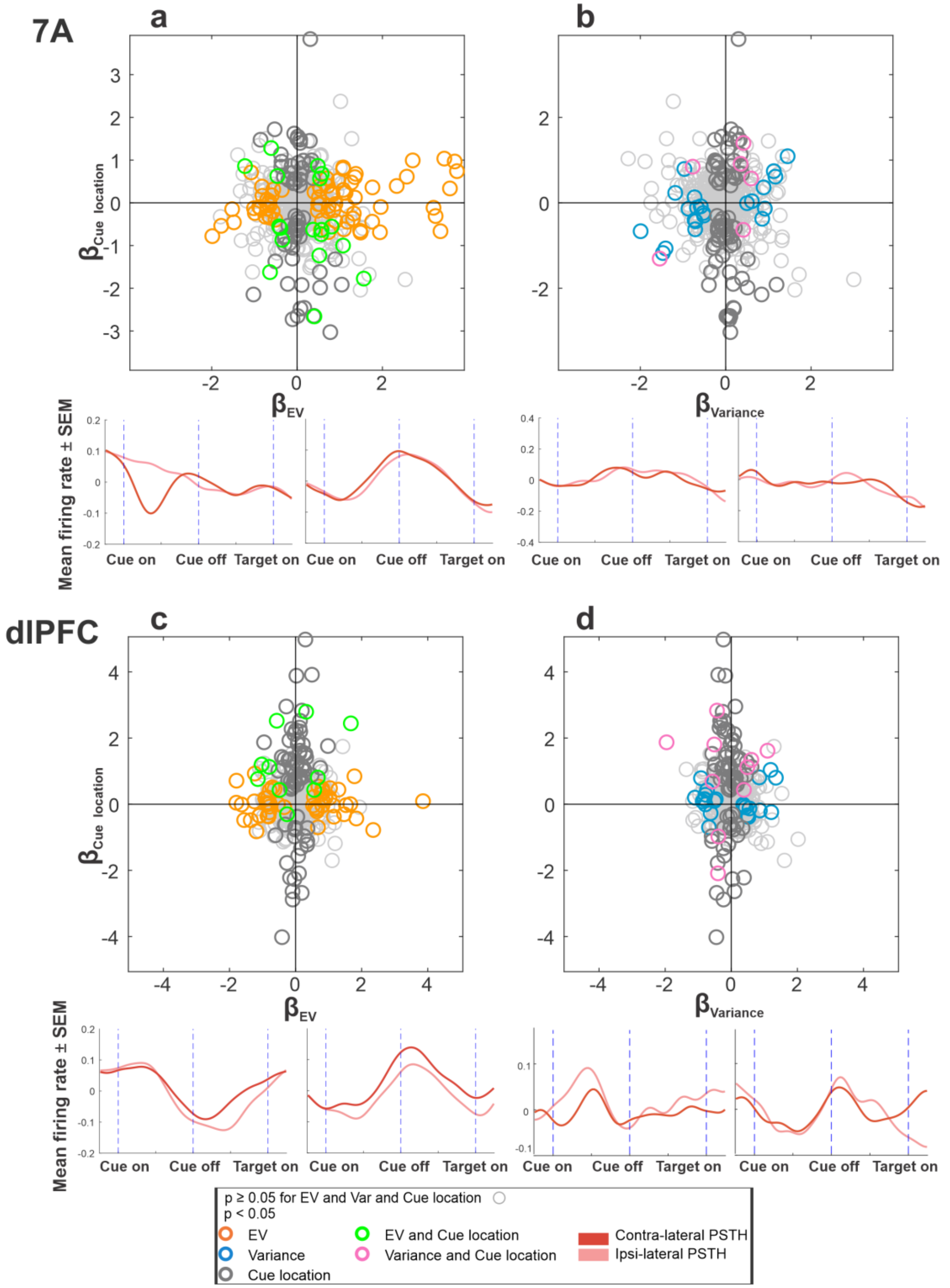

For each area (7A, **a** and **b**; dlPFC, **c** and **d**), the scatter plots compare GLM coefficients for the cue location ($\beta_{Cue\ location}$, ordinate) with coefficients of EV (β_{EV}, left panel, abscissa) and variance ($\beta_{Variance}$, right panel, abscissa). Points are individual neurons color-coded according to the significance of their main effects as indicated in the legend. The PSTHs below show the firing rates for cells with significant positive or negative sensitivity to variance or EV, including the few cells that are also sensitive to cue location. The traces are sorted by cue location (dark red: contralateral to the recording hemisphere).

Across the population of all cells there was a bias towards contralateral encoding in dlPFC (mean ± SEM coefficients 0.134 ± 0.035; $p = 25\times10^{-5}$, Wilcoxon signed-rank test relative to 0) but not in 7A (mean ± SEM coefficients -0.059 ± 0.032; p = 0.42). However, cue location encoding was not correlated with either variance or EV encoding (Spearman correlation, all p>0.06). The PSTHs reveal no consistent spatial selectivity in the variance or EV sensitive cells and importantly, no consistent short-latency visual response to cue onset, confirming the independence of reward and spatial selectivity.

Fig. S3: Decoding accuracy for congruent and incongruent train-testing regimes.

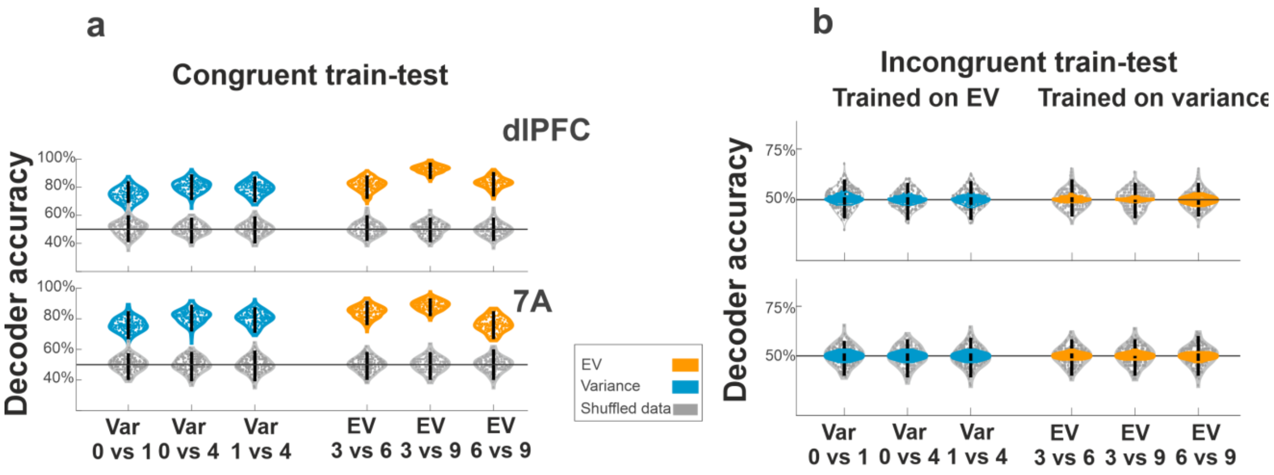

(a) Incongruent regime. Each violin plot shows the test accuracy scores for binary classification of EV (orange) or variance levels (blue) for classifiers trained on the same variable. Each violin plot shows the accuracy over 200 bootstrap iterations from the original dataset (colored) and the dataset with randomized labels (gray). The whiskers in each distribution show 95% confidence interval around the mean. Accuracy is above chance if the CI do not include zero and do not overlap with the randomized distributions. **(b) Incongruent regime.** Same as in **a**, but for classifiers trained and tested on the other variable.

Fig. S4: SFC is not consistently modulated by EV across areas, or by variance or EV within area.

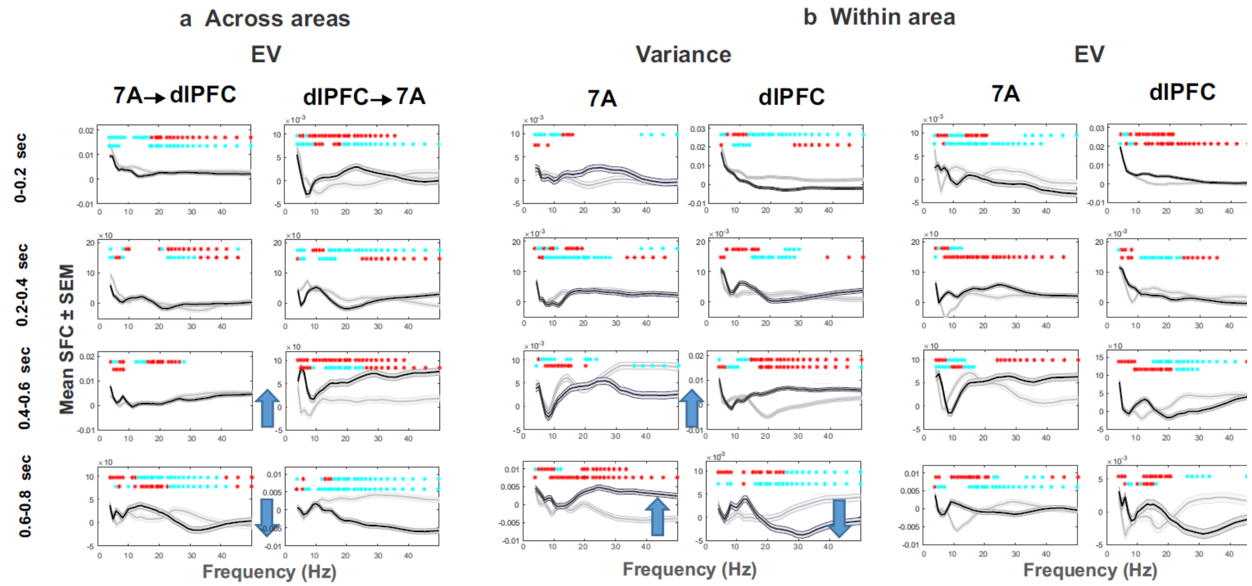

From top to bottom, rows show successive 200 ms intervals aligned on cue onset. Blue arrows show intervals in which both monkeys showed significant modulations of the same sign. In **a**, this included an increase followed by decrease in dlPFC to 7A SFC in response to EV in, respectively, the early and late memory periods. In **b**, it included an increase followed by decrease in within-dlPFC SFC in response to variance in, respectively, the early and late memory periods. All other conventions as in **Fig 7b**.

Fig. S5: LFP-LFP coherence. No consistent modulation with variance or EV.

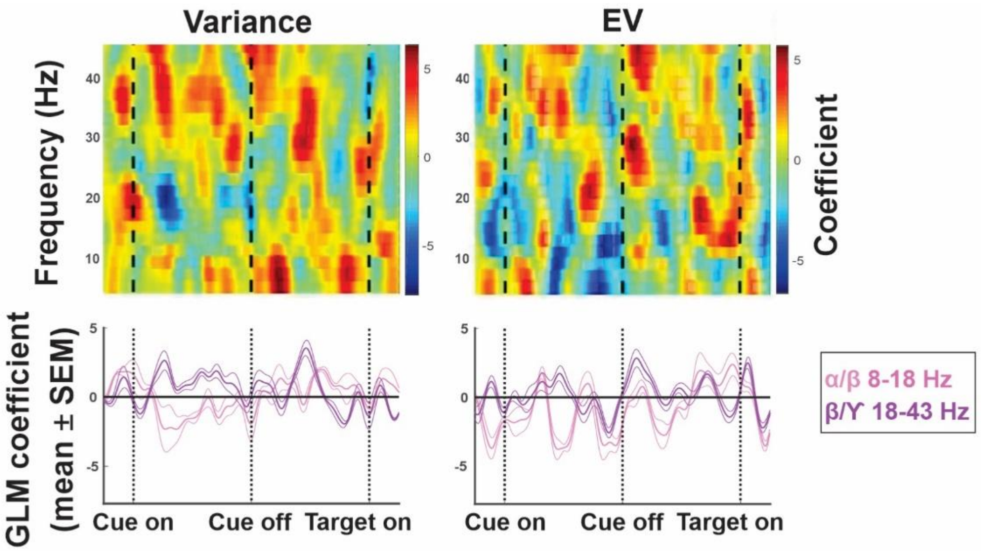

The degree to which two areas synchronize their oscillations is a sensitive index of neural connectivity. We therefore tested whether, and how, the coherence of oscillations between frontal and parietal LFPs was affected by variance and EV. In every session, for different time and frequencies (1 ms time bins and 2 Hz frequency bins), Weighted Phase Lag Index (WPLI; debiased WPLI option in the ft_connectivityanalysis() function of the FieldTrip toolbox) of LFP was calculated across trials and all electrode pairs between dlPFC and 7A. GLM with factors of EV, variance and their interaction was then fitted to the coherence maps from different sessions, assuming normal distribution and identity link function. There was no consistent strong modulation of coherence with variance or EV.

Fig. S6: Risk attitude and relative sensitivity to variance and EV

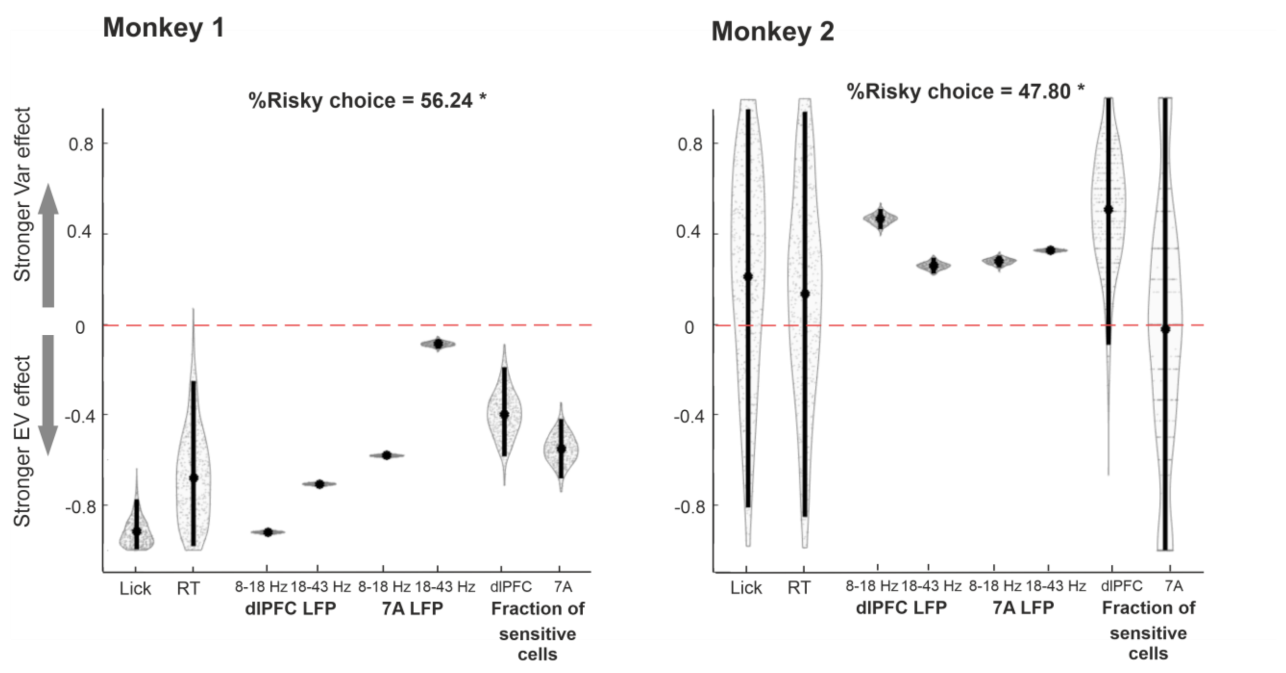

The panels show contrast indices (y-axis) capturing the relative sensitivity to variance and EV for several behavioral and neural measures. For licking, reaction time (RT) and LFP power we used the GLM coefficients (β) indicating the strength of each effect (for the LFP, we took the mean coefficient in every ROI) and defined the contrast index as $\frac{|\beta_{Var}| - |\beta_{EV}|}{|\beta_{Var}| + |\beta_{EV}|}$. Note that, because the GLMs used standardized regressors, the coefficient magnitudes can be directly compared. For the fraction of sensitive cells, the index was $\frac{|F_{Var}| - |F_{EV}|}{|F_{Var}| + |F_{EV}|}$ where F_{EV} and F_{Var} indicate fraction of EV and variance sensitive cells. All indices thus range between -1 and 1, with positive and negative values indicating, respectively, stronger sensitive to variance or EV. Error bars show 95% confidence intervals estimated from 500 bootstraps across trials for RT and licking, across time-frequency samples within the ROIs for LFP, and across cells for the fraction of sensitive cells. Violin plots show the bootstrap samples.

To study risk preference, we conducted a choice version of the task in which the monkeys were given two reward cues randomly placed on either side of the central fixation point. Upon the disappearance of the fixation point, the monkeys were free to choose one of the cues by making a saccade to it, and received a reward drawn from the distribution signaled by the chosen cue. We analyzed 6,346 independent choice trials from monkey 1 and 5,977 independent trials from monkey 2.

The %risky choice (the fraction of trials in which the monkey selected the higher variance cue; title of each panel) indicated that monkey 1 was risk seeking and monkey 2 was risk averse (* indicates $p < 0.014$, signed-rank test relative to 50%). A 2-way ANOVA showed that this was a highly significant individual difference at all levels of EV ($p < 10^{-9}$ for effect of monkey; $p > 0.14$ for monkey x EV interaction). Moreover, this individual difference corresponded with a higher relative sensitivity to variance in monkey 2 (who was risk-averse) relative to monkey 1 (who was risk seeking) that was evident in licking, RT, LFP power and fraction of selective cells. We found no equivalent modulation of the SFC.
