# Supplementary material for: Reward uncertainty asymmetrically affects information transmission within the monkey fronto-parietal network
Source: Commun Biol. 2020 Oct 21;3:594. doi: 10.1038/s42003-020-01320-6 (PMC7578031; doi:10.1038/s42003-020-01320-6)
Supplement: Supplementary file 2 — Description of Additional Supplementary Files [file 42003_2020_1320_MOESM2_ESM.pdf]

Description of Additional Supplementary Files

File Name: Supplementary Data 1
Description: Source data for Fig 1d.

File Name: Supplementary Data 2
Description: Source data for Fig 2.

File Name: Supplementary Data 3
Description: Source data for Fig 3. The excel file includes four sheets corresponding to the PSTHs and Line plots from dlPFC and 7A.

File Name: Supplementary Data 4
Description: Source data for Fig 4. The excel file includes two sheets corresponding to the Fig 4a and Fig 4b.

File Name: Supplementary Data 5
Description: Source data for Fig 5.

File Name: Supplementary Data 6
Description: Source data for Fig 6, insets. The excel file includes two sheets corresponding to the areas.

File Name: Supplementary Data 7
Description: Source data for Fig 7. The excel file includes two sheets corresponding to the Fig 7 a-b and Fig 7 c-f.
