# Supplementary material for: Reward uncertainty asymmetrically affects information transmission within the monkey fronto-parietal network
Source: Commun Biol. 2020 Oct 21;3:594. doi: 10.1038/s42003-020-01320-6 (PMC7578031; doi:10.1038/s42003-020-01320-6)
Supplement: Supplementary file 10 — Reporting summary [file 42003_2020_1320_MOESM10_ESM.pdf]

Corresponding author(s): Jacqueline Gottlieb

Last updated by author(s): May 8, 2020

Reporting Summary

Nature Research wishes to improve the reproducibility of the work that we publish. This form provides structure for consistency and transparency in reporting. For further information on Nature Research policies, see our Editorial Policies and the Editorial Policy Checklist.

Statistics

For all statistical analyses, confirm that the following items are present in the figure legend, table legend, main text, or Methods section.

n/a	Confirmed	
☐	☒	The exact sample size (n) for each experimental group/condition, given as a discrete number and unit of measurement
☐	☒	A statement on whether measurements were taken from distinct samples or whether the same sample was measured repeatedly
☐	☒	The statistical test(s) used AND whether they are one- or two-sided<br>*Only common tests should be described solely by name; describe more complex techniques in the Methods section.*
☐	☒	A description of all covariates tested
☐	☒	A description of any assumptions or corrections, such as tests of normality and adjustment for multiple comparisons
☐	☒	A full description of the statistical parameters including central tendency (e.g. means) or other basic estimates (e.g. regression coefficient) AND variation (e.g. standard deviation) or associated estimates of uncertainty (e.g. confidence intervals)
☐	☒	For null hypothesis testing, the test statistic (e.g. F, t, r) with confidence intervals, effect sizes, degrees of freedom and P value noted<br>*Give P values as exact values whenever suitable.*
☒	☐	For Bayesian analysis, information on the choice of priors and Markov chain Monte Carlo settings
☒	☐	For hierarchical and complex designs, identification of the appropriate level for tests and full reporting of outcomes
☐	☒	Estimates of effect sizes (e.g. Cohen's d, Pearson's r), indicating how they were calculated

Our web collection on statistics for biologists contains articles on many of the points above.

Software and code

Policy information about availability of computer code

Data collection	MonkeyLogic was used to present and monitor the task; Cereplex software was used for neuronal recording; WaveSorter was used for spike sorting.
Data analysis	MATLAB 2016b was used to write the custom code. Fieldtrip and Chronux software packages were used for data analysis.

For manuscripts utilizing custom algorithms or software that are central to the research but not yet described in published literature, software must be made available to editors and reviewers. We strongly encourage code deposition in a community repository (e.g. GitHub). See the Nature Research guidelines for submitting code & software for further information.

Data

Policy information about availability of data

All manuscripts must include a data availability statement. This statement should provide the following information, where applicable:

- Accession codes, unique identifiers, or web links for publicly available datasets
- A list of figures that have associated raw data
- A description of any restrictions on data availability

The data recorded and analyzed in this study are available from the corresponding author upon reasonable request.

Field-specific reporting

Please select the one below that is the best fit for your research. If you are not sure, read the appropriate sections before making your selection.

☒ Life sciences ☐ Behavioural & social sciences ☐ Ecological, evolutionary & environmental sciences

For a reference copy of the document with all sections, see nature.com/documents/nr-reporting-summary-flat.pdf

Life sciences study design

All studies must disclose on these points even when the disclosure is negative.

Sample size	No statistical methods were used to pre-determine sample sizes. Two animal subjects were sued in this study, which is similar to the sample size in most previous publications. In total, 522 cells in 7A and 531 cells in dlPFC were analyzed from the two animals, which is within the range of the average sample sizes reported in previous publications that used multi-channel electrode arrays.
Data exclusions	Neurons were selected based on pre-defined, standard, electrophysiological activity within the trial, as outlined in the manuscript. This ensures including only those cells which are active during the trial, not those which are only active between trials and become silent as the behavioral task begins. For every LFP channel, trials in which the LFP signal had poor quality were excluded, as outlined in the manuscript, to minimize the effect of hardware noise and artefacts. Error trials were excluded from the analysis as a pre-established way to focus on trials of interest.
Replication	Results were replicated in two animals.
Randomization	Experimental conditions were randomized in every session to eliminate any bias due to predictive activities in the neural data. Single cells and LFPs were sampled in every session as a random sample from each area.
Blinding	Investigators were not blinded. Blinding is irrelevant, because the results are based on statistical analyses, the exclusion criteria are separate from the phenomena being investigated, clearly reported, and standard in the field.

Reporting for specific materials, systems and methods

We require information from authors about some types of materials, experimental systems and methods used in many studies. Here, indicate whether each material, system or method listed is relevant to your study. If you are not sure if a list item applies to your research, read the appropriate section before selecting a response.

Materials & experimental systems

n/a	Involved in the study	
☒	☐	Antibodies
☒	☐	Eukaryotic cell lines
☒	☐	Palaeontology and archaeology
☐	☒	Animals and other organisms
☒	☐	Human research participants
☒	☐	Clinical data
☒	☐	Dual use research of concern

Methods

n/a	Involved in the study	
☒	☐	ChIP-seq
☒	☐	Flow cytometry
☒	☐	MRI-based neuroimaging

Animals and other organisms

Policy information about studies involving animals; ARRIVE guidelines recommended for reporting animal research

Laboratory animals	Two adult male rhesus monkeys (Macaca mulatta; 9-12kg)
Wild animals	Wild animals were not used in this study.
Field-collected samples	Field samples were not used in this study.
Ethics oversight	All methods were approved by the Animal Care and Use Committees of Columbia University and New York State Psychiatric Institute as complying with the guidelines within the Public Health Service Guide for the Care and Use of Laboratory Animals.

Note that full information on the approval of the study protocol must also be provided in the manuscript.
